# Anxiety Disorder amongst Secondary School Children in an Urban City in Nigeria

**Published:** 2010-09

**Authors:** Angela I. Frank-Briggs, E. A. D. Alikor

**Affiliations:** *Department of Paediatrics, University of Port Harcourt Teaching Hospital, Port Harcourt, Negeria*

**Keywords:** anxiety, depression, fear, urban schools, children

## Abstract

Anxiety is a source of concern to the clinicians as it is co morbid with other mental disorders, particularly depression and learning disabilities, and it causes low self-esteem. The aim of this research was to evaluate the prevalence of anxiety disorder amongst secondary school children in Port Harcourt. A two-staged stratified sampling method was used to select the schools. Structured questionnaire based on Vanderbilt ADHD Diagnostic Teacher Rating Scale for anxiety and depression symptoms was used in evaluating the students. The questionnaires administered to the students were filled with the assistance of the researchers and the classroom teachers. Direct verbal interview was conducted for those noted to have symptoms of any of the various types of anxiety disorders and fears. Out of 885 students, 91 met the criteria for the diagnosis of anxiety/ depression disorder; prevalence was 10.28%, age range was 9-18 years. There were 37 males and 54 females giving a male: female ratio of 0.69:1. Majority 52 (57.14%) of the children lived with their parents, 28 (30.77%) of them lived with family relations and 11 (12.09%) of them were working as house helps to other families. The reasons given for being anxious were poor self image, fear of death, repeated physical and sexual abuses by their care givers and other adults. Learning disability was the major associated co morbid disorder (18.68%). Generalized anxiety was the most common type of anxiety disorder identified (32.97%). Anxiety disorders are debilitating chronic conditions. When it affects school aged children it contributes significantly to poor academic performance.

## INTRODUCTION

Anxiety disorders are the most common group of psychiatric illnesses in children (1, 2). Anxiety is a blanket term covering several different forms of abnormal and pathological fear. Anxiety disorders are often debilitating chronic conditions, which can be present from an early age or begin suddenly after a triggering event (2). The disorder is frequently accompanied by physiological symptoms such as headache, excessive sweating, muscle spasms, palpitations, and hypertension, which in some cases lead to fatigue and exhaustion. Those affected can also flare up at times of high stress.

Although in casual discourse the words anxiety and fear are often used interchangeably, in clinical practice, they have distinct meanings. Anxiety is defined as an unpleasant emotional state for which the cause is either not readily identified or perceived to be uncontrollable and unavoidable; whereas, fear is an emotional and physiological response to a recognized external threat. The term anxiety disorder, however, includes fears (phobias) as well as anxieties (3).

Anxiety disorders are often co morbid with other mental illnesses, particularly clinical depression, which may occur in as many as 60% of people with anxiety disorder (4). There is considerable overlap between symptoms of anxiety and depression, and the same environmental triggers can provoke symptoms in either condition, this may help to explain this high rate of co morbidity (3).

It is also known that anxiety disorder is more likely to occur among those with a positive family history of the disorder (5). Children and adolescents with anxiety disorder typically experience intense fear, worry, or uneasiness that can last for a long period of time and significantly affect their everyday activity. If not treated early, anxiety disorder can lead to repeated school absenteeism and inability to complete ones education, this is usually due to poor concentration and maladjustment. Some affected children have impaired relationship with their peers, others have low self-esteem. Failure to identify and manage the disorder could also result in alcoholism and use of other hard drugs by affected persons. In some individuals the disorder continues into adult life (5).

A survey conducted in the United States found that as many as 18% of Americans may be affected by one or more of the different types of the anxiety disorder (6). Kashani and Orvasche reported a prevalence rate of 17.3% amongst adolescents in their study (7). There is paucity of information on anxiety disorder among children in Port Harcourt metropolis, this formed the basis for carrying out this study.

## MATERIALS AND METHODS

The study was conducted in primary schools in Port Harcourt between 5^th^ January to 30^th^ June 2009. Port Harcourt is a metropolitan city, the capital of Rivers State and one of the Niger Delta states of Nigeria. A two-staged stratified sampling method was used to select the schools in the Port Harcourt metropolis. The schools were first stratified based on location. Final selection was done by a simple random sampling method, balloting from each sub-section of the strata. The study was carried out by utilizing a structured questionnaire (See Appendix A) and another questionnaire based on Vanderbilt ADHD Diagnostic Teacher Rating Scale for anxiety and depression symptoms (8). Diagnosis required three or more counted behaviors from questions 29-35 (See Appendix B). Nine hundred and fifty students from four different schools were recruited into the study. The questionnaires administered to the students were filled with the assistance of the researchers and the classroom teachers. Direct face-to-face verbal interview was conducted by the researchers for those noted to have symptoms of anxiety disorders. Direct questioning was done to confirm the type of anxiety disorder, reason for the anxiety and associated comorbidities. The term report cards for the students were sighted to ascertain their academic performance. This was done for all the children. Those of them with anxiety disorder were given psychotherapy by a child psychologist and special counseling sessions were arranged for them.

## RESULT

There were 885 students were studied and 91 of them met the criteria for the diagnosis of anxiety/ depression disorder, giving a prevalence of 10.28%.

### Demographic Data

The age range of the entire students ranged from 9-18 years with a modal age of 13 years. Sex distribution of those with the anxiety disorder showed that 37 were males and 54 were females giving a male: female ratio of 0.69:1.

### Others results

All the children were resident in Port Harcourt metropolis. Majority 52 (57.14%) lived with their parents, 28 (30.77%) of them lived with family relations and 11 (12.09%) of them were working as house helps to other families. The various reasons for anxiety are shown on Table 1. The reasons included being anxious about physical appearance or self image, anxiety and fear of witchcraft activities, fear of repeated physical and sexual abuses by some of their care givers and strangers in their neighbourhood. A number of co morbid disorders were associated with the anxiety disorder; this is shown on Table 2.

**Table 1:** Showing reasons for anxiety in the pupils

Reasons	Number	Percentage

Poor academic performance	31	34.06
Self image	15	16.48
Imaginary creatures		
Ghost	13	14.29
Witches	10	10.99
Death	07	7.7
Accident	04	4.4
Abuse		
Physical	07	7.7
Sexual	02	2.2
Unknown	02	2.2

**Table 2:** Showing comorbid disorders amongst the pupils

Disorder	Number	Percentage

Epileptic seizure	17	18.68
Learning disorder	15	16.48
Mathematics		
Writing		
ADHD	6	6.59
Conduct disorder	4	4.40
NIL	49	53.85

ADHD, Attention Deficit Hyperactivity Disorder.

It was noticed that the academic performance of the children was poor. Their previous term’s result showed that their overall average ranged between 28-46%, none of them had up to 50% in their class academic activity.

Table 3 shows the different types of anxiety disorder the students suffered. Generalized anxiety disorder was the most common type.

**Table 3:** Showing types of anxiety disorders in the 91 students

Type	Number	Percentage

Generalized AD	30	32.97
Separation AD	24	26.38
Phobias		
Schools	08	8.79
Unfamiliar places	05	5.49
Body image	05	5.49
Kidnap	03	3.30
Post Traumatic		
Sexual abuse	11	12.09
Physical abuse	5	5.49

AD, Anxiety Disorder.

## DISCUSSION

Anxiety disorders are very common during the childhood/adolescence period and because of its chronicity, severity and comorbid problems associated with it, children need to be identified early, and necessary treatment initiated to limit the deleterious effects on their mental and emotional functions (9).

The different types of anxiety disorders and fears that affected these children greatly impaired their quality of life. This has also been reported in other studies (10, 11). Those affected with generalized anxiety disorder engaged in extreme, unrealistic worry about everyday life activities. They worried unduly about their physical appearance, their academic performance was poor, their interaction with people was not cordial, and they were not usually at their best in performing domestic chores. This resulted in frequent verbal and physical assaults from their teachers and caregivers. Typically, these young people were very self-conscious and felt tensed and embarrassed when being interviewed. These behaviors have been reported previously in another studies (10, 12). It was also noted that about 33% of the children suffered from separation disorder. Those affected admitted that that they were often sad, withdrawn, depressed and had intense fear.

Children and adolescents with phobias have unrealistic and excessive fears of certain situations or objects (13). Many phobias have specific names, and the disorder usually centers on being fearful of animals, storms, water, heights, and other difficult situations. Our study showed that about 23% of children and adolescents had social phobias. Those affected were afraid of being left with strangers in unfamiliar places especially those that had been victims of kidnap and rape. These children exhibited intense fear, they were withdrawn and unfriendly. Some other children also expressed that they were unduly worried about their body image (obesity). These obese children were terrified of being criticized and / or mocked by their classmates and other adults. The children with phobias usually avoided people and situations they feared, making the disorder greatly restrict their lifestyle.

Another type of anxiety disorder was the post-traumatic stress disorder noted in 18% of our study group. Children and adolescents can develop post-traumatic stress disorder after they experience a very stressful event. Such events may include experiencing physical or sexual abuse, being a victim of or witnessing violence, or living through a disaster, such as a bombing or earthquakes (14). The children in our study who had post-traumatic stress were small in number. They were victims of physical and sexual abuse. They were shy, moody and very reluctant to narrate their ordeal. The small number may be because some others may be unwilling to discuss their ugly experience following abuse of this nature. Young people with post-traumatic stress disorder experience the event repeatedly through strong memories, flashbacks, or other kinds of troublesome thoughts (14, 15). As a result, they may try to avoid anything associated with the trauma. School refusal and truancy were common with those students; similar problems have also been documented in other studies (15, 16). Such children overreact when startled and in addition, they may have difficulty sleeping. The ugly experience may prevent these children from developing more optimistic and realistic appraisal of life.

## CONCLUSION

It is known that anxiety disorders, if left unidentified and untreated, may persist and lead to adult psychological problems, and may be a risk factor in the development of co- morbid child psychopathology, such as mood disorders and behavioral problems (17, 18). Furthermore, anxiety has been associated with general social problems such as negative self-image, dependency on adults in social situations, comparatively poor problem-solving skills, unpopularity and low rates of interaction with peers. It is therefore recommended that, there is need for early intervention and prevention programs for anxiety disorders in young people. Early intervention program (19-21), which will include availability of efficacious programs, ethical issues, planning, implementation and evaluation of interventions will go a long way in reducing the morbidity associated with anxiety disorders.

## APPENDIX A

### Questionnaire on Anxiety Disorder

1. Name ___________________________
2. Age ____________________________
3. Sex_______________Male ( ) Female ( )
4. Class____________________________
5. Address__________________________
6. Who do you live with__________Parents ( ) Guardian ( ) Others (specify)
7. Are you worried, fearful or anxious about any thing Yes ( ) No ( )
8. If yes, what is it, specify______________
9. Have you ever been assaulted in your life? Yes ( ) No ( )
10. Mention the type of assault ___________
11. What was your class average in the last terms examination?
12. Do you have learning difficulty in any subject, if yes, specify____________________________________
13. Do you have any psychiatry illness or epilepsy or any other illness?
14. If yes to question 13, specify type of illness. _________________________________________________________________________________
15. Type of anxiety disorder (to be determined by the researcher). __________________________________________________________________________________

## APPENDIX B

### INSTRUCTIONS AND SCORING

Behaviors are counted if they are scored 2 (often) or 3 (very often).

**Inattention.** Requires six or more counted behaviors from questions 1-9 for indication of the predominantly inattentive subtype.

**Hyperactivity/impulsivity.** Requires six or more counted behaviors from questions 10-18 for indication of the predominantly hyperactive/impulsive subtype.

**Combined subtype.** Requires six or more counted behaviors each on both the inattention and hyperactivity/impulsivity dimensions.

**Oppositional defiant and conduct disorders.** Requires three or more counted behaviors from questions 19-28.

**Anxiety or depression symptoms.** Requires three or more counted behaviors from questions 29-35.

The performance section is scored as indicating some impairment if a child scores 1 or 2 on at least one item.

### Vanderbilt ADHD Diagnostic Teacher Rating Scale

Name: __________________________
Grade: __________________________
Date of Birth: _____________________
Teacher: _________________________
School: __________________________

Each rating should be considered in the context of what is appropriate for the age of the children you are rating.

### Frequency Code: 0, Never; 1, Occasionally; 2, Often; 3, Very Often

1. Fails to give attention to details or makes careles mistakes in schoolwork (0 1 2 3);
2. Has difficulty sustaining attention to tasks or activities (0 1 2 3);
3. Does not seem to listen when spoken to directly (0 1 2 3);
4. Does not follow through on instruction and fails to finish schoolwork (not due to oppositional behavior or failure to understand) (0 1 2 3);
5. Has difficulty organizing tasks and activities (0 1 2 3);
6. Avoids, dislikes, or is reluctant to engage in tasks that require sustaining mental effort (0 1 2 3);
7. Loses things necessary for tasks or activities (school assignments, pencils, or books) (0 1 2 3);
8. Is easily distracted by extraneous stimuli (0 1 2 3);
9. Is forgetful in daily activities (0 1 2 3);
10. Fidgets with hands or feet or squirms in seat (0 1 2 3);
11. Leaves seat in classroom or in other situations in which remaining seated is expected (0 1 2 3);
12. Runs about or climbs excessively in situations in which remaining seated is expected (0 1 2 3);
13. Has difficulty playing or engaging in leisure activities quietly (0 1 2 3);
14. Is “on the go” or often acts as if “driven by a motor” (0 1 2 3);
15. Talks excessively (0 1 2 3);
16. Blurts out answers before questions have been completed (0 1 2 3);
17. Has difficulty waiting in line (0 1 2 3);
18. Interrupts or intrudes on others (e.g., butts into conversations or games) (0 1 2 3);
19. Loses temper (0 1 2 3);
20. Actively defies or refuses to comply with adults’ requests or rules (0 1 2 3);
21. Is angry or resentful (0 1 2 3);
22. Is spiteful and vindictive (0 1 2 3);
23. Bullies, threatens, or intimidates others (0 1 2 3);
24. Initiates physical fights (0 1 2 3);
25. Lies to obtain goods for favors or to avoid obligations (i.e., “cons” others) (0 1 2 3);
26. Is physically cruel to people (0 1 2 3);
27. Has stolen items of nontrivial value (0 1 2 3);
28. Deliberately destroys others’ property (0 1 2 3);
29. Is fearful, anxious, or worried (0 1 2 3);
30. Is self-conscious or easily embarrassed 0 1 2 3;
31. Is afraid to try new things for fear of making mistakes (0 1 2 3);
32. Feels worthless or inferior (0 1 2 3);
33. Blames self for problems, feels guilty (0 1 2 3);
34. Feels lonely, unwanted, or unloved; complains that “no one loves him/her” (0 1 235) Is sad, unhappy, or depressed (0 1 2 3).

### PERFORMANCE

#### Problematic Average above average

**Academic Performance**

1. Reading (1 2 3 4 5)
2. Mathematics (1 2 3 4 5)
3. Written expression (1 2 3 4 5)

**Classroom Behavioral Performance**

1. Relationships with peers (1 2 3 4 5)
2. Following directions/rules (1 2 3 4 5)
3. Disrupting class (1 2 3 4 5)
4. Assignment completion (1 2 3 4 5)
5. Organizational skills (1 2 3 4 5)

**Frequency Code: 0, Never; 1, Occasionally; 2, Often; 3, Very Often.**
